# Prevalence, phylogenomic insights, and phenotypic characterization of *Salmonella enterica* isolated from meats in the Tamale metropolis of Ghana

**DOI:** 10.1002/fsn3.1647

**Published:** 2020-05-22

**Authors:** Frederick Adzitey, Gabriel Ayum Teye, Daniel Gyamfi Amoako

**Affiliations:** ^1^ Department of Veterinary Science University for Development Studies Tamale Ghana; ^2^ Department of Animal Science University for Development Studies Tamale Ghana; ^3^ Infection Genomics and Applied Bioinformatics Division, Antimicrobial Research Unit University of KwaZulu‐Natal Durban South Africa

**Keywords:** antibiotics, characterization, meat, phylogenomic analysis, *Salmonella* species

## Abstract

Characterization of foodborne pathogens including *Salmonella* species allows for the determination of their relationship and/or relatedness with others. This study characterized *Salmonella enterica (S. enterica)* isolated from five meat types (mutton, beef, chevon, guinea fowl, and local chicken) obtained from Tamale metropolis, Ghana. The *S. enterica* were characterized phenotypically (*n* = 44) based on their antibiotic resistance pattern with the disc diffusion method and genetically (*n* = 16) using whole‐genome sequencing (WGS) as well as with bioinformatic analysis for the prediction of their clonal and phylogenomic relationship. Of the 225 meat samples examined, 107 (47.56%) were positive for *S. enterica*. Mutton was the most contaminated meat type and the least was local chicken. The 44 *S. enterica* isolates exhibited five different antibiotic patterns with multiple antibiotic resistance (MAR) index ranging from 0.13 to 0.63. Resistant to only erythromycin was most common and was exhibited by 34 isolates (77.27%). Four isolates were resistant to four different antibiotics (TeAmpSxtECro) with a percentage of 9.09%, while two isolates (4.55%) were resistant to none of the antibiotics. The sequenced *S. enterica* isolates consisted of 7 serovars and 8 clonal lineages with the *S. enterica subsp. enterica serovar Hato* (ST5308) being the predominate strain. Phylogenomic analysis showed that the isolates clustered according to their serovars and sequence types (clonal lineages). However, further metadata insights coupled with the phylogenomics revealed a complex intraspread of multiple *S. enterica subsp. enterica serovars* in diverse meat sources in areas in Tamale which is very worrying for infection management. In summary, our study provides useful insights into *S. enterica* in meat reservoirs obtained from Tamale metropolis, Ghana.

## Graphical abstract

Meat samples were contaminated by *Salmonella* and aerobic bacteria. *Salmonella* species exhibited some resistance to antibiotics. The *Salmonella* isolates were genetically diverse.

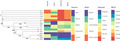

## 1 INTRODUCTION

Meats are major component of human diets and serve as an excellent source of protein. Other nutrients including fats (omega‐3‐ polyunsaturated fatty acids), minerals (iron, magnesium, potassium, selenium sodium, and zinc), and vitamins (vitamin A, vitamin E, B6, B12, niacin, thiamine, and riboflavin) can also be found in meats (Ahmad, Imran, & Hussain, 2018; America Meat Science Association, 2016). They are consumed worldwide by all races except vegetarians and people who have deliberately refuse to eat meats due to welfare concerns and/or love for animals. The consumption of meats has been associated with the risk of foodborne infections and illnesses.

Salmonellae are gram‐negative facultative anaerobe bacteria that have been associated with foodborne infections (Wallace & Hammack, 2013). For instance, a recent foodborne outbreak suspected to be caused by *Salmonella* Dublin was linked to the consumption of ground beef (Centers for Disease Control & Prevention, 2019). Other Salmonella infections have been linked to pork, 30% hospitalizations with 0 death (Centers for Disease Control & Prevention, 2015), mechanically separated chicken, 22% hospitalizations with 0 death (Centers for Disease Control & Prevention, 2014) and chicken, 33% hospitalizations with 0 death (Centers for Disease Control & Prevention, 2013). A total of 91,662 human confirmed cases of *Salmonella* infections were reported in 2017 by the European Union, with 43.1% hospitalizations and 0.25% case fatality (European Food Safety Authority, 2018). Saba et al. (2013) indicated that data on *Salmonella* infections in most developing and underdeveloped countries is scared.

Similarly, reported incidences of *Salmonella* infections in Ghana are limited if not unavailable, but the organism has been found in various meats including, beef, chevon, mutton, and pork (Adzitey, 2015; Adzitey, Nsoah, & Teye, 2015; Adzitey, Teye, & Anachinaba, 2015; Danikuu, 2004). The treatment of *Salmonella* infections relies on the use of antibiotics. Meanwhile, resistance of *Salmonella* to antibiotics is a treat to public health and a concern worldwide. *Salmonella* isolated from various meat samples have been demonstrated to be resistant to one or more antibiotics such as amoxycillin/clavulanic acid, ampicillin, chloramphenicol, sulfamethoxazole/trimethoprim, tetracycline, vancomycin, and others (Adzitey, 2015; Arslan & Eyi, 2010; Ejo, Garedew, & Alebachew, 2016).

Characterization of foodborne pathogens has some importance including the determination of their history, relationship, and closeness. This intend helps to predict their characteristics, properties, or behavior from others. Characterization of foodborne pathogens at the phenotypic and genotypic levels have been achieved using serotyping, antibiotic profiling, whole‐genome sequencing, multilocus sequencing typing, pulsed‐field gel electrophoresis, repetitive extragenic palindromic, among others (Adhikari et al., 2010; Adzitey, Deli, & Ali, 2015; Adzitey, Saba, & Deli, 2014; Jaja, Bhembe, Green, Oguttu, & Muchenje, 2019).

Report on the characterization and comparison of *Salmonella* from various meat sources (mutton, beef, chevon, guinea fowl, and local chicken) in the Tamale metropolis is scare. Therefore, this study was carried out to characterize *Salmonella enterica* isolated from various meat types using antibiotic resistance, and phylogenomic analyses.

## 2 MATERIALS AND METHODS

### 2.1 Study area

The study was conducted in the Tamale metropolis, Ghana. The metropolis lies in between latitude 9°16 and 9°34 North and longitudes 0°36 and 0°57 West (Ghana Statistical Service, 2014). The Tamale metropolis shares boundaries with Sanarigu District to the west and north, Mion District to the east, East Gonja to the south, and Central Gonja to south‐west. It is the capital town of the Northern Region of Ghana and the third most populace town in Ghana (Ghana Statistical Service, 2014).

### 2.2 Samples examined

Two hundred and twenty‐five (225) samples made of beef, chevon, mutton, local chicken, and guinea fowl were examined. Forty‐five (45) samples each of the various meat types were randomly sampled from both traditional and close modern markets between the hours of 10:00–14:00 GMT. An area of 10 cm^2^ was swabbed using sterile cotton swabs. The swab samples were transported in an ice chest containing ice block and were analyzed immediate on reaching the laboratory.

### 2.3 Analysis of meat samples for *Salmonella enterica*

A slightly modified method of the Food and Drug Administration‐Bacteriological Analytical Manual was used (Adzitey, Nsoah, et al., 2015; Wallace & Hammack, 2013). Briefly, swab samples were pre‐enriched in 10 ml Buffered Peptone Water (BPW) and incubated at 37°C for 24 hr. Then, 0.1 ml aliquots were transferred into 10 ml Rappaport and Vassiliadis (RV) and Selenite Cystine (SC) broths. Samples in RV broths were incubated at 42°C for 24 hr while samples in SC broths were incubated at 37°C for 24 hr (enrichment). After which 0.1 ml of the aliquots were streaked on Xylose Lysine Deoxycholate and Brilliant Green agars and incubated at 37°C for 24–48 hr. Presumptive *Salmonella* colonies were picked, purified, Gram stained and subjected to the following biochemical tests; growth characteristics on triple sugar iron, lysine iron and Simon citrate agars, and urease production. *Salmonella* isolates were confirmed by Latex Agglutination Kit for *Salmonella* (Oxoid Limited). All media used were also purchased from Oxoid Limited.

### 2.4 Analysis of meat samples for microbial load

Microbial load determination was done as describe by Maturin and Peeler (2001) and Adzitey, Ekli, and Abu (2019). Swabs were dipped into 10 ml of 1% BPW and thoroughly agitated. Serial dilutions (10^−1^–10^−5^) were made in 9 ml BPW using 1 ml, and 100 µl dilution spread plated onto Plate Count Agar (PCA; Oxoid Limited) plates. The plates were incubated at 37°C for 24 hr and colonies counted using a colony counter.

### 2.5 Phenotypic antibiotic susceptibility testing

The disk diffusion method of Bauer, Kirby, Sherris, and Turk (1966) was used for antibiotic susceptibility testing of 44 pure *S. enterica* isolates against the following antibiotics; ampicillin (10 μg), ceftriaxone (30 μg), chloramphenicol (30 μg), ciprofloxacin (5 μg), erythromycin (15 µg), gentamicin (10 μg), suplfamethoxazole/trimethoprim (22 μg), and tetracycline (30 μg). The purified *Salmonella* species were inoculated in Trypticase Soy Broth (TSB; Oxoid Limited) and incubated at 37°C for 18 hr. The turbidity was adjusted to 0.5 McFarland standard using sterile TSB and spread plated on Müller Hinton Agar (MHA; Oxoid). Four antibiotic disks were placed on the surface of the MHA at a distance to avoid overlapping of inhibition zones. The plates were incubated at 37°C for 24 hr. After incubation, the inhibition zones were measured, and the results interpreted using the Clinical Laboratory Standard Institute (2017). Multiple antibiotic resistance (MAR) index was calculated and interpreted according to Krumperman (1983) using the formula: a/b, where “a” represents the number of antibiotics to which a particular isolate was resistant and “b” the total number of antibiotics tested.

### 2.6 Genomic sequence, assembly annotation and bioinformatic analysis of *Salmonella enterica*

Sixteen *S. enterica* were randomly selected, sequenced, assembled, and annotated as described by Tay et al. (2019).

### 2.7 WGS‐based molecular typing and phylogenomic analyses of *Salmonella enterica*

Multilocus sequence typing (MLST) was performed in silico using the WGS data online platform MLST v2.0 (https://cge.cbs.dtu.dk/services/MLST/) from the assembled genomes which also predicted the allelic profiles of the seven housekeeping genes of *S. enterica*. The reference Salmonella online platform, SeqSero v1.0 (www.denglab.info/SeqSero) was used to infer the serotypes of the isolates.

A phylogenetic tree was also constructed for all the genomes to determine the relatedness of the *S. enterica* strains using CSI Phylogeny‐v1.4 (https://cge.cbs.dtu.dk/services/CSIPhylogeny/), an online service which identifies SNPs from WGS data, filters and validates the SNP positions, and then infers phylogeny based on concatenated SNP profiles. A bootstrapped with 100 replicates indicator was applied to identify recombined regions and provide the phylogenetic accuracy in groups with little homoplasy. The Figtree was used to edit and visualize the phylogenetic tree. The phylogeny was visualized alongside annotations for isolate demographics (source and area of collection) and WGS in silico molecular typing (serovar and sequence type) metadata using Phandango (Hadfield et al., 2017).

### 2.8 Accession numbers

The raw read sequences and the assembled whole‐genome contigs have been deposited in GenBank under the project number PRJNA484344.

### 2.9 Statistical analysis

Data obtained for *S. enterica* was analyzed using binary logistic generalized linear model of Statistical Package for Service Solutions Program version 20.0; and test for statistical difference was done using wald chi‐square. Means were separated at 5% significancet level. Microbial load was analyzed using GenStat Release 12 Edition; and Analysis of Variance was used to test the significant difference at *p* < .05.

## 3 RESULTS

### 3.1 Distribution of *Salmonella enterica* and microbial load in the various meat types

The distribution of *S. enterica* and microbial load in the meats is shown in Table 1. *S. enterica* were found in beef 19 (42.22%), chevon 22 (48.89%), guinea fowl 20 (44.44%), local chicken 13 (28.89%), and mutton 33 (73.33%). The contamination of mutton by *S. enterica* was significantly higher (*p* < .05) than the rest of the meat types. The presence of *S. enterica* in local chicken was significantly lower (*p* < .05) than chevon but not guinea fowl and beef. Contamination of beef, chevon, and guinea fowl by *S. enterica* did not differ (*p* > .05) from each other.

**TABLE 1:** Distribution of *Salmonella enterica* and microbial load in various meat types sold at the Tamale metropolis, Ghana

Sample	No. of samples examined	No. (%) positive	Microbial load (log cfu/cm^2^)
Beef	45	19 (42.22)	3.36
Chevon	45	22 (48.89)	4.03
Guinea fowl	45	20 (44.44)	3.33
Local chicken	45	13 (28.89)	4.34
Mutton (Lamb)	45	33 (73.33)	4.9
Overall	225	107 (47.56)	3.99

Abbreviations: No., number of samples positive for *Salmonella enterica*.

The microbial load was 3.36, 4.03, 3.33, 4.34, and 4.90 log cfu/cm^2^ for beef, chevon, Microbial load did not differ significantly (*p* = .212) among the various meat samples.

### 3.2 Phenotypic antibiotic susceptibility testing of *Salmonella enterica* isolates

The phenotypic antibiotic characterization of the 44 *S. enterica* isolates is shown in Tables 2 and 3. The *S. enterica* isolates were highly resistant to erythromycin (93.18%) but susceptible to ampicillin (79.55%), ciprofloxacin (97.73%), chloramphenicol (93.18%), gentamicin (79.55%), suplfamethoxazole/trimethoprim (90.91%), and tetracycline (84.09%).

**TABLE 2:** Phenotypic antibiotic susceptibility of *Salmonella enterica* in various meat types sold at the Tamale metropolis, Ghana

Antimicrobial	% Resistance	% Intermediate resistance	% Susceptibility
Ampicillin (AMP) 10 µg	11.36	9.09	79.55
Ciprofloxacin (CIP) 5 µg	0.00	2.27	97.73
Ceftriaxone (CRO) 30 µg	13.64	20.45	65.91
Chloramphenicol (C) 30 µg	0.00	6.82	93.18
Erythromycin (E) 15 µg	93.18	0.00	6.82
Gentamicin (CN) 10 µg	4.55	15.91	79.55
Sulphamethoxazole/trimethoprim (SXT) 22 µg	9.09	0.00	90.91
Tetracycline (TE) 30 µg	9.09	6.82	84.09
Overall %	17.61	7.67	74.72

**TABLE 3:** Antibiotic resistance profile and multiple antibiotic resistance index of individual *Salmonella enterica* isolated from various meat types sold at the Tamale metropolis, Ghana

No.	Salmonella code	Source	Antibiotic resistant profile^a^	Number of antibiotics	MAR index
1	NB10	Beef	AmpECro	3	0.38
2	AB11	Beef	Cro	1	0.13
3	AC2	Chevon	E	1	0.13
4	AC3	Chevon	E	1	0.13
5	AC5	Chevon	E	1	0.13
6	AM2	Mutton	E	1	0.13
7	AM3	Mutton	E	1	0.13
8	NC2	Chevon	E	1	0.13
9	NC6	Chevon	E	1	0.13
10	CB1	Beef	E	1	0.13
11	CB5	Beef	E	1	0.13
12	CB14	Beef	E	1	0.13
13	CC3	Chevon	E	1	0.13
14	CC5	Chevon	E	1	0.13
15	CC8	Chevon	E	1	0.13
16	CM1	Mutton	E	1	0.13
17	CM7	Mutton	E	1	0.13
18	CM11	Mutton	E	1	0.13
19	NC13	Chevon	E	1	0.13
20	NM1	Mutton	E	1	0.13
21	CG1	Guinea fowl	E	1	0.13
22	CG4	Guinea fowl	E	1	0.13
23	CG15	Guinea fowl	E	1	0.13
24	NLC9	Local chicken	E	1	0.13
25	NLC13	Local chicken	E	1	0.13
26	SG14	Guinea fowl	E	1	0.13
27	TG5	Guinea fowl	E	1	0.13
28	TG14	Guinea fowl	E	1	0.13
29	TG15	Guinea fowl	E	1	0.13
30	NLC8	Local chicken	E	1	0.13
31	SLC10a	Local chicken	E	1	0.13
32	SLC10b	Local chicken	E	1	0.13
33	SLC10c	Local chicken	E	1	0.13
34	TLC7a	Local chicken	E	1	0.13
35	TLC7b	Local chicken	E	1	0.13
36	TLC7c	Local chicken	E	1	0.13
37	AB7	Beef	ECn	2	0.25
38	AC3	Chevon	ECn	2	0.25
39	AM10	Mutton	None	0	0.00
40	NB8	Beef	None	0	0.00
41	NB2	Beef	TeAmpSxtECro	5	0.63
42	NM7	Mutton	TeAmpSxtECro	5	0.63
43	NM14	Mutton	TeAmpSxtECro	5	0.63
44	SG15	Guinea fowl	TeAmpSxtECro	5	0.63

Note

^a^ Ampicillin (Amp) 10 μg; ciprofloxacin (Cip) 5 µg; ceftriaxone (Cro) 30 µg; gentamicin (Cn) 10 µg; erythromycin (E) 15 µg; sulphamethoxazole/trimethoprim (Sxt) 22 µg; tetracycline (Te) 30 µg.

The multiple antibiotic (MAR) index ranged from 0.13 (resistant to one antibiotic) to 0.63 (resistant to five antibiotics). The 44 *S. enterica* isolates were resistance to zero (4.55%), one (79.55%), two (4.55%), three (2.27%), and five (9.09%) antibiotics. They exhibited five different resistance patterns thus, AmpECro (1 isolate), Cro (1 isolate), E (34 isolates), ECn (2 isolates), and TeAmpSxtECro (4 isolates). Multidrug resistant that is resistant to 3 or more different classes of antibiotics was observed in five (11.36%) of the isolates.

### 3.3 Phylogenomic analysis and metadata insights

**FIGURE 1:**
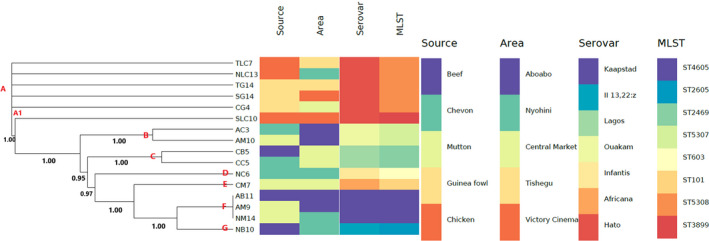
The whole‐genome MLST phylogenetic branch and metadata (WGS in‐silico molecular typing [serovar and sequence type] and Demographics [source and area]) coupled using Phandango in isolated *Salmonellae enterica* strains (*n* = 16) isolated from meats in the Tamale metropolis of Ghana

The phylogenetic relationship and epidemiological distribution of the *S. enterica* isolate from meat samples in Tamale are depicted in Figure 1. The isolates generally clustered according to their serovars and sequence types (clonal lineages). Of note, there were 7 clades (grouped A–G) and 1 subclade (A1, ST3899) which collaborated with the 8 clonal lineages. This affirms the ability of WGS‐based typing methods to accurately differentiate between isolates using appropriately curated databases.

## 4 DISCUSSION

Results from this study indicated that, meat samples obtained from the Tamale metropolis were contaminated with *S. enterica*. Therefore, eating undercooked meats can serve as sources of *Salmonella* infections. Contamination of meats by *S. enterica* was highest in mutton and least in local chicken. This is not surprising since it was observed during sample collection that the environment where local chickens were processed was neater than where mutton and other ruminants were processed. Cattle, sheep, and goats are normally processed into beef, chevon, and mutton, respectively, in the Tamale abattoir which lacks all the equipment required for a modern abattoir. Furthermore, butchers slaughtered animals on the floor and do not adhere to strict hygienic slaughter and personal hygiene. Guinea fowls and local chickens are normally processed by individuals in an open market or by the roadside. Most often, guinea fowls and local chickens are sold directly after processing at the processed point, while beef, chevon, and mutton are transported to from the abattoir to the market for sale. They are transported from the abattoir to the market by taxis, motor kings®, motorbikes, bicycles, and sometimes meat vans that are not well maintained. The meats are transported openly or covered with plastic rubber. The selling points are either at the roadside, in traditional open or close modern markets. All of these expose meats to microorganisms blow by dust, wind, or smoke from cars. The contamination of the various meat types by *S. enterica* can also be attributed to the tables on which meats are placed for sale and the knives used for cutting meats (Adzitey, Nsoah, et al., 2015). The *S. enterica* could have cross‐contaminated the meats from the gastrointestinal tracts. Adzitey, Sulleyman, and Kum (2020) also found that meat sellers in the Tamale metropolis do not sterilize their knives and majority (48%) of them sold meat on open tables, which expose meats to flies, dusts, smoke from vehicles, and other contaminants. The contamination of beef samples by *Salmonella* species was higher (75%) in the Techiman municipality (Adzitey, Nsoah, et al., 2015) and lower (31%) in the Tamale metropolis (Adzitey, 2015) of Ghana as compared to this study. Arslan and Eyi (2010) found *Salmonella* species in 29.3% of poultry meat and 16% of beef samples in Bolu, Turkey. The prevalence results for poultry meat were similar to this study but a higher prevalence of *Salmonella* was detected in beef samples in this study. In Gondar, Ethiopia, *Salmonella* species were detected in 12% of raw meat which was lower than that of this study (Ejo et al., 2016).

The intrinsic characteristics of meats such as nutrient composition, pH, water activity, and temperature promote the growth of microorganisms. Meat is a good medium for the growth of microorganisms including *S. enterica* because it rich in protein, lipids, and other nutrients which microorganisms use for their growth (Prescott, Harley, & Klein, 2002). *Salmonella* spp. have also been reported to grow at a temperature range of 5°C–47°C (optimum of 35°C–37°C), pH range of 4–9 (optimum of 6.5–7.5) and a water between of 0.99 and 0.94 (Dodd, Aldsworth, Stein, Cliver, & Riemann, 2017). Meat has a neutral pH and a water activity of between 0.98 and 0.99 (Meter Food, 2017). The pH and water activity of meat favors the growth of *S. enterica*. Also the meats sampled were warm and sold under ambient temperature, thus providing favorable environment for the growth of *S. enterica* and other microorganisms. The microbial load (<10^6^) observed in this study is considered satisfactory (Center for Food Safety, 2014). Numerically, it was highest in mutton and lowest in guinea fowl. The scalding of guinea fowl could have contributed to the lower microbial load observed as compared to mutton. Microbial load for local chicken was also expected to be lower than that of beef and chevon but this was not observed. The presence of microbial load in the meat samples examined means that lapses occurred during the processing of the meat samples as the muscle of a healthy living animal is essentially sterile. A higher microbial load ranging from 3.99–6.19 log cfu/cm^2^ in fresh guinea fowls (Adzitey, Teye, et al., 2015) and 4.75–6.31 log cfu/cm^2^ for fresh beef (Anachinaba, Adzitey, & Teye, 2015) was reported in the Bolgatanga municipality, Ghana. Soepranianondo, Wareham, Budiarto, and Diyantoro (2019) reported a lower microbial load of 1.62 log cfu/g in beef samples collected from slaughterhouses in East Java, Indonesia as compared to this study. However, a higher microbial load (5.40–8.35 log cfu/g) in comparison with this study was reported by Jahan, Mahbub‐E‐Elahi, and Siddique (2015) in beef obtained from markets of Sylhet Sadar in Banladesh.

The resistance of *S. enterica* to some of the antibiotics used in this study can be attributed to the use of these antibiotics for the treatment of cattle, goats, guinea fowls, local chickens, and sheep during their production. Reports from other researchers have shown that farm animals are sources of antibiotic resistant *Salmonella* strains (Founou, Amoako, Founou, & Essack, 2018; Jaja et al., 2019; Nair, Venkitanarayanan, & Johny, 2018; Wang et al., 2019) that can be transferred to humans. Adzitey, Nsoah, et al. (2015) also found that *Salmonella* species of beef origin were highly resistant to Erythromycin (75.56%), but susceptible to ciprofloxacin (100%), gentamicin (86.67%), ceftriaxone (73.33%), and sulfamethoxazole/trimethoprim (68.89%). Arslan and Eyi (2010) reported that *Salmonella* species of poultry and beef origin were susceptible to ampicillin (63.6 vs. 0.0), ciprofloxacin (81.8 vs. 100), chloramphenicol (59.1 vs. 91.7), gentamicin (72.7 vs. 91.7), sulfamethoxazole/trimethoprim (63.6 vs. 91.7), and tetracycline (31.8 vs. 58.3), respectively.

Similarly, to the current study Adzitey, Nsoah, et al. (2015) reported MAR index range of 0.11–0.67 for *Salmonella* species isolated from beef. They also found that the *Salmonella* species exhibited multiple antibiotic resistance and 23 different resistance patterns. Jaja et al. (2019) reported MAR index of 0.67–0.93 and 0.60–0.93 for *Salmonella* species isolated from meats collected from formal meats sector and informal slaughter points, respectively. Other researchers including Arslan and Eyi (2010) and Ejo et al. (2016) have also reported multidrug resistance *Salmonella* strains of meat origin. Arslan and Eyi (2010) indicated that 62% of *Salmonella* strains exhibited multiple resistance to three or more antimicrobial agents. Ejo et al. (2016) showed that 20% *Salmonella* isolates were resistant to one antimicrobial, while 80% were resistant to two or more antimicrobials. Jaja et al. (2019) found a high prevalence of multidrug‐resistant *S. enterica* isolates in meats and indicated that there is a high risk associated with the consumption of contaminated meat. Resistant *Salmonella* species can contaminate carcasses, processing equipment, and other foods which pose a risk for public and animal health.

The tree analysis coupled with metadata revealed useful insights with regard to the diversity of serovars clones in meat sources and area of collection (Figure 1). For instance; meat sources; beef, chevon, and mutton contained different serovars of *S. enterica* isolates which were clonally distinct (Figure 1). This finding corroborated with other studies reported worldwide specifically in Europe (Müller, Jansen, Grabowski, & Kehrenberg, 2018), Africa (Thomas et al., 2020), and Asia (Yang et al., 2019) which isolated different serovars *S. enterica* in food samples. However, all the guinea fowl and chicken samples contained the same serovar; *S. enterica subsp. enterica serovar Hato* strain which predominately belonged to the ST5308 clone (Figure 1). Interestingly, Fagbamila et al. (2017) also reported the presence of Hato serovar in chicken layer farms in Nigeria.

With respect to the area of collection; there was clonal spread of the *S. enterica subsp. enterica serovars* in some areas in Tamale irrespective of the meat source. For example; the Kaapstad serovar (ST4605) was found in both beef and mutton samples in Aboabo while the Ouakam serovar (ST5307) were also isolated in chevon and mutton in the same area. More so, beef and chevon from the central market area contained the *S. enterica subsp. enterica serovar Lagos* (ST2469) strain. A similar trend was also observed in guinea fowl and chicken from both Victory Cinema and Tishegu area. This complex intraspread of multiple *S. enterica subsp. enterica serovars* in diverse meat sources in areas in Tamale is very worrying for infection management. A combination of WGS data, demographics, and graphical visualization should be applied to offer vital insights and increase confidence during molecular epidemiological investigations (Amoako et al., 2019).

## 5 CONCLUSION

Overall, 107 (47.56%) *Salmonella species* and 3.99 log cfu/cm^2^ microbial load were detected in the meat samples. Mutton (lamb) was the most contaminated source. Phenotypic characterization revealed a high resistance to erythromycin but susceptibility (≥90) to ciprofloxacin, chloramphenicol, and sulfamethoxazole/trimethoprim. Phylogenomic analysis showed that the isolates clustered according to their serovars and sequence types (clonal lineages). However, further metadata insights coupled with the phylogenomics revealed a complex intraspread of multiple *S. enterica subsp. enterica serovars* in diverse meat sources in areas in Tamale which is very worrying for infection management. In summary, our study provides useful insights into *S. enterica* in meat reservoirs obtained from Tamale metropolis, Ghana which warrants an urgent action to curb this possible threat.

## CONFLICT OF INTEREST

The authors declare that they have no conflict of interest.

## ETHICAL APPROVAL

The study did not involve any human or animal testing.

## INFORMED CONSENT

Verbal consent was obtained from all meat sellers.
